# Preparation and Characterization of Mo Doped in BiVO_4_ with Enhanced Photocatalytic Properties

**DOI:** 10.3390/ma10080976

**Published:** 2017-08-21

**Authors:** Bitao Liu, Xuelian Yan, Hengqing Yan, Yucen Yao, Yanhua Cai, Jumeng Wei, Shanyong Chen, Xuhui Xu, Lu Li

**Affiliations:** 1 Chongqing Key Laboratory of Environmental Materials and Remediation Technology, Research Institute for New Materials Technology, Chongqing University of Arts and Sciences, Yongchuan, Chongqing 402160, China; liubitao007@163.com (B.L.); yanxl0902@163.com (X.Y.); yanhengqing@163.com (H.Y.); yaoyucen@126.com (Y.Y.); caiyh651@aliyun.com (Y.C.); 2 College of Chemistry and Materials Engineering, Anhui Science and Technology of University, Fengyang 233100, China; 3 School of Materials Science and Engineering, Kunming University of Science and Technology, Kunming 650093, China; yuyu6593@126.com

**Keywords:** BiVO_4_, MoO_3_, photocatalyst, visible light

## Abstract

Molybdenum (Mo) doped BiVO_4_ was fabricated via a simple electrospun method. Morphology, structure, chemical states and optical properties of the obtained catalysts were characterized by X-ray diffraction (XRD), scanning electron microscopy (SEM), transmission electron microscopy (TEM), X-ray photoelectron spectroscopy (XPS), UV-vis diffuse reflectance spectroscopy (DRS), N_2_ adsorption–desorption isotherms (BET) and photoluminescence spectrum (PL), respectively. The photocatalytic properties indicate that doping Mo into BiVO_4_ can enhance the photocatalytic activity and dark adsorption ability. The photocatalytic test suggests that the 1% Mo-BiVO_4_ shows the best photocatalytic activity, which is about three times higher than pure BiVO_4_. Meanwhile, 3% Mo-BiVO_4_ shows stronger dark adsorption than pure BiVO_4_ and 1% Mo-BiVO_4_. The enhancement in photocatalytic property should be ascribed to that BiVO_4_ with small amount of Mo doping could efficiently separate the photogenerated carries and improve the electronic conductivity. The high concentration doping would lead the crystal structure transformation from monoclinic to tetragonal phase, as well as the formation of MoO_3_ nanoparticles on the BiVO_4_ surface, which could also act as recombination centers to decrease the photocatalytic activity.

## 1. Introduction

Semiconductor photocatalysis based on solar energy has been demonstrated to be one of effective techniques to eliminate organic pollutants in wastewater [1]. Conventional TiO_2_ is considered as an efficient UV light-driven photocatalyst. However, the UV light is only 4% in the solar light spectrum, thereby limiting its application [2]. In this regard, researchers have focused on exploring new photoactive materials, which could be driven by considerable and cost-efficiently visible light. Monoclinic bismuth vanadate (BiVO_4_) with narrow band gap (Eg = 2.4 eV) is one of the excellent visible-light-driven photocatalysts [3]. Theoretically, its solar-to-hydrogen conversion efficiency can reach up to 9.2%. However, the actual photocatalytic efficiency of pristine BiVO_4_ is far below what is expected, because it suffers from poor electronic transportation and short carrier diffusion length, resulting in numerous bulk recombination of electron–hole pairs [4,5]. Therefore, effectively inhibiting the recombination of photo-induced electron–holes is the key factor to improve the photocatalytic activity of monoclinic BiVO_4_.

By the incorporation with inorganic nanostructures, such as Bi_2_WO_6_ [6], MoS_2_ [7] and g-C_3_N_4_ [8], the photocatalytic activity can be efficiently improved. However, the synthesis process of such composite is usually complex. Another useful way to increase the photocatalytic activity is doping metal/non-metallic elements into catalyst, which can easily modify its electronic properties and thus enhance the photocatalytic activity. In general, doping could extend the optical absorption, enhance the surface adsorption for dyes molecules and improve the electrical conductivity [9]. Various studies have shown that the incorporation of molybdenum (Mo) into BiVO_4_ can bring out significant improvement in electronic properties (e.g., carrier concentrations or CB level) [4,10]. For example, Parmar and coworkers [9] concluded that the estimated carrier concentration of Mo-doped BiVO_4_ photoanode was about two times higher than that of undoped BiVO_4_. The VBs and CBs of Mo-doped BiVO_4_ were broadened because of the overlapped orbitals. Ding et al. [11] stated that the substitute of Mo^6+^ in V^5+^ sites could facilitate the separation of carriers. However, all of the experimental studies and theoretical calculations were based on photocatalytic water-splitting. The limitation of the systematic research on the structure and morphology evolution can hinder the improvement of the photocatalytic degradation of organic pollutants, especially for the BiVO_4_.

In this paper, we report a simple and good repeatability method to fabricated Mo doped BiVO_4_. The application in dye degradation for MB under visible light was performed. The details of the photocatalytic properties are also discussed.

## 2. Experimental and Characterization

### 2.1. Synthesis of BiVO_4_ and Mo-Doped BiVO_4_

All chemicals used in this work are of analytical-reagent grade without further purification. The procedure for spinning precursor with same stoichiometric ratio of bismuth and vanadium source is followed by our previous paper [12]. Then, different amounts of ammonium molybdate (0.4, 1, 1.5, 3 mol %) were added into these solutions, while stirring for 12 h to obtain homogeneous precursors. During the electrospinning process, the electrospun temperature was fixed at 60 °C with 15 kV voltages. The collected composites were first dried in a vacuum oven at 70 °C for 40 min followed by calcination at 450 °C for 2 h with a heating rate of 1 °C/min.

### 2.2. Characterization

Powder X-ray diffraction was carried on TD-3500 X-ray diffractometer (Tongda, Dandong, Liaoning, China) using Cu Kα radiation, operating at 30 kV and 20 mA. An ESCA Lab MKII (VG, London, UK) was employed for X-ray photoelectron spectroscopy, to investigate surface electronic properties of BiVO_4_ and Mo-doped BiVO_4_. Scanning electron microscope (SEM, S-4800, Hitachi, Tokyo, Japan) and high-resolution transmission electron microscopy (HRTEM,) were taken with a JEM-2010 (JEOL, Tokyo, Japan) operating at 20 kV. The surface area (BET) was measured on a BELSORT-max (MicrotracBEL, Osaka, Japan) instrument. The UV-vis diffuse reflectance spectra (DRS) of the materials were obtained on a UV-vis spectrophotometer with an integrating sphere (U-3900, Hitachi, Tokyo, Japan). Photoluminescence spectra were registered on OmniPL-LF325 spectrofluorometer (Zolix, Beijing, China) with 325 nm laser as a radiation source.

### 2.3. Photocatalytic Test and PEC Measurements

During the photodegradation process, 0.05 g of the catalyst was suspended in 50 mL of methylene blue (MB) solution with the concentration of 10 ppm. After stirring for 30 min in dark to establish the adsorption–desorption equilibrium, the solution was illuminated to visible light with a cut filter of 420 nm using 500 W Xe arc lamp as light source, under continuous stirring. At a given time interval, 4 mL of suspension were taken out and centrifuged for 10 min to remove the photocatalyst. The centrifuged solution was analyzed by recording the variations of the absorption peak at 664 nm using a UV-vis spectrometer (Cary 50, Varian, Shanghai, China).

Electrochemical tests were performed on an AUTOLAB PGSTAT302N station (Methrohm, Herisau, Switzerland). A saturated calomel electrode (SCE) and Pt wire were used as reference and counter electrode, respectively. ITO glasses covered with 2 mg of the prepared powders were used as work electrode. A 500 W Xe arc lamp with a 420 nm cutoff filter was employed as visible light source. The photoanode current was measured in 0.1 M Na_2_SO_4_ electrolyte, with a fixed applied potential of 0.0 V. Electrochemical impedance spectra (EIS) were measured in 1 M KOH electrolyte.

## 3. Results and Discussion

**Figure 1:**
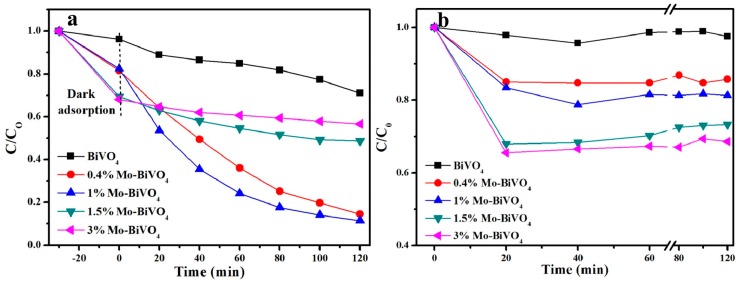
(**a**) Photocatalytic degradation of MB using undoped BiVO_4_ and different amount of Mo-doped BiVO_4_ under visible light (λ > 420 nm) illumination; and (**b**) adsorption properties of the five products above.

The photocatalytic degradation for MB solution is shown in Figure 1a. In dark adsorption process, it is clearly seen that all Mo-BiVO_4_ samples exhibit stronger adsorption property for MB molecules compared with the undoped BiVO_4_. Of note, dark adsorption performance of 1.5% Mo-BiVO_4_ (30.66%) and 3% Mo-BiVO_4_ (31.93%) is higher than that of 0.4% Mo-BiVO_4_ (18.36%) and 1% Mo-BiVO_4_ (17.48%). However, the 1.5% Mo-BiVO_4_ and 3% Mo-BiVO_4_ show a lower photocatalytic activity. After 120 min illumination, photocatalytic activity order of different catalysts is as follows: 1% Mo-BiVO_4_ (88.57%) > 0.4% Mo-BiVO_4_ (85.46%) > 1.5% Mo-BiVO_4_ (51.32%) > 3% Mo-BiVO_4_ (43.35%) > BiVO_4_ (29%).

Additional adsorption test in dark was measured to evaluate the MB degradation during the above irradiation process. As presented in Figure 1b, the concentration of MB solution is observably decreased within the first 20 min for all five samples. In addition, the adsorption performance order for the five samples is in accordance with the above. In the next 100 min, the concentration of MB solution for five samples is almost equal to the results of first 20 min.

Based on above analysis, it is indicated that suitable doping of Mo would be the key point to enhance the photocatalytic activity or dark adsorption ability. In view of similar photocatalytic properties of 0.4% Mo-BiVO_4_ and 1% Mo-BiVO_4_ (as well as 1.5% Mo-BiVO_4_ and 3% Mo-BiVO_4_), we choose BiVO_4_, 1% Mo-BiVO_4_ and 3% Mo-BiVO_4_ for further characterization, and then investigate the effect of Mo-doping on the photocatalytic activity for BiVO_4_.

**Figure 2:**
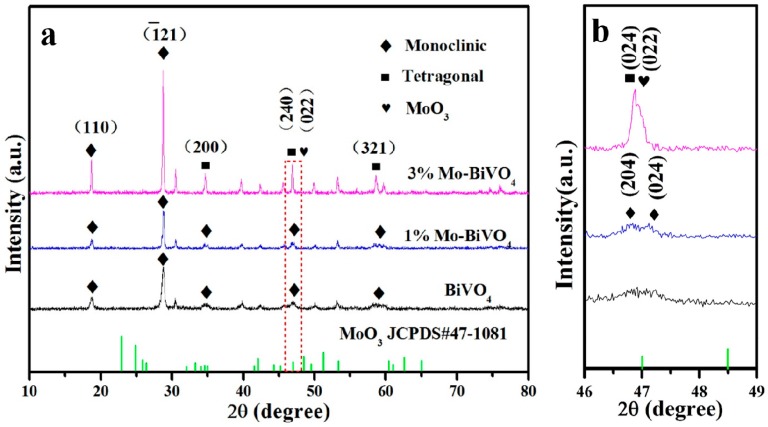
(**a**) XRD patterns of undoped BiVO_4_, 1% Mo-BiVO_4_ and 3% Mo-BiVO_4_; (**b**) the magnified XRD peaks at 47° of all samples.

**Figure 3:**
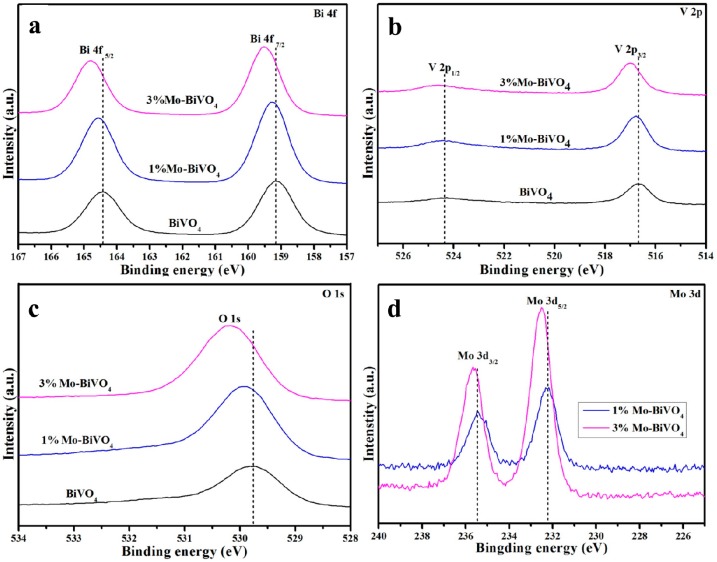
High-resolution XPS spectra of undoped BiVO_4_, 1% Mo-BiVO_4_ and 3% Mo-BiVO_4_: (**a**) Bi 4f; (**b**) V 2p; (**c**) O 1s; and (**d**) Mo 3d.

Figure 2 presents the typical XRD patterns of bare BiVO_4_ and Mo-BiVO_4_ samples. The BiVO_4_ and 1% Mo-BiVO_4_ show relatively broad peaks and low intensity, indicating their low crystallinity. The 3% Mo-BiVO_4_ exhibits sharp peaks with strong intensity because of their high crystallinity (Figure 2a) [13], indicating that higher doping concentration could promote the grain growth and lead to an increased crystallinity. Numerous studies have demonstrated that appropriate crystal defects such as surface oxygen vacancies could act as scavengers for electrons or holes, promoting the separation efficiency of carriers [1,14,15]. Especially, the peaks of BiVO_4_ and 1% Mo-BiVO_4_ are well indexed to monoclinic structure (JCPDS NO.14-0688). The 3% Mo-BiVO_4_ structure, however, progresses toward a mixture of monoclinic and tetragonal phase, which can be observed from the sharp characteristic tetragonal peaks located at 34.5°, 46.9°, and 58.6° (JCPDS NO.48-0744). Similar results have also been found by Bard A.J. and coworkers [16]. Tetragonal structure BiVO_4_ could decrease the photocatalytic activity due to its regular electronic arrangement, which was consistent with the photocatalytic test results. The phase transformation in 1.5% Mo-BiVO_4_ results in different photocatalytic performance compared to 1% Mo-BiVO_4_. Figure 2b shows the magnified XRD peaks between 46° and 49°. It can be clearly seen that two peaks at 47° could be observed. These two peaks are well indexed to (024) and (022) planes for tetragonal BiVO_4_ and MoO_3_ (JCPDS NO. 47-1081), respectively. The XPS technique was employed to investigate the surface electronic properties of samples. The high-resolution XPS spectral of BiVO_4_, 1% Mo-BiVO_4_ and 3% Mo-BiVO_4_ samples are presented in Figure 3. The element spectral clearly indicates typical spin-orbit doublet separation except for O 1s. For bare BiVO_4_, two main asymmetric peaks at 159.2 and 164.4 eV are ascribed to Bi 4f_7/2_ and Bi 4f_5/2_, which are the features of Bi^3+^ in BiVO_4_, and the two asymmetric peaks at 516.7 and 524.3 eV are corresponded to V 2p_3/2_ and V 2p_1/2_, which should be ascribed to the V^5+^ in BiVO_4_ [9,17]. For two Mo-BiVO_4_ samples, there is a gradual shift toward higher binding energies in the characterized peaks of Bi 4f, V 2p, and O 1s with the increase of Mo concentration. The specific binding energies of elements are listed in Table 1. The slightly higher binding energies of all ions (Bi^3+^, V^5+^ and O^2−^) in Mo-doped BiVO_4_ samples are mainly associated with the relatively higher electronegativity of the dopants (Mo^6+^: 2.16 > V^5+^: 1.63) [9]. As for the 3% Mo-BiVO_4_, the binding energies of Mo^6+^ is still higher than 1% Mo-BiVO_4_. Combined with the XRD results, it is concluded that this phenomenon should be ascribed to the existence of MoO_3_ on BiVO_4_ surface. The Mo concentration can be calculated from XPS data. The atomic ratio of Mo:V increase with increasing the Mo doping (from 1.76%:8.78% to 4.47%:8.95%), indicating the success doping.

**Figure 4:**
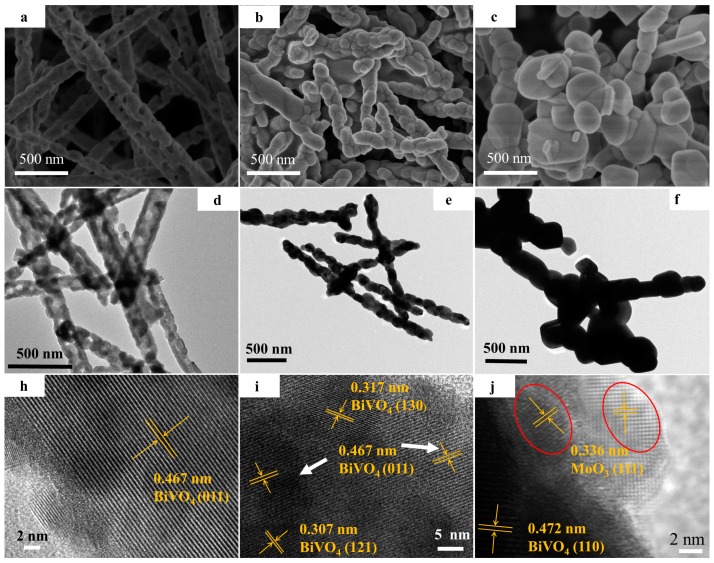
SEM (**a**–**c**); TEM (**d**–**f**) and HRTEM (**h**–**j**) images of BiVO_4_, 1% Mo-BiVO_4_ and 3% Mo-BiVO_4_, respectively.

**Figure 5:**
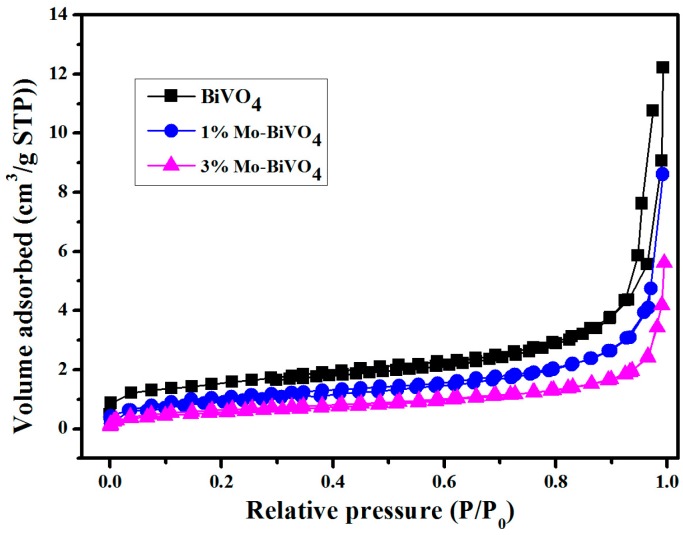
N_2_ adsorption–desorption isotherms of BiVO_4_, 1% Mo-BiVO_4_ and 3% Mo-BiVO_4_.

It is generally accepted that the morphologies greatly influence the photocatalytic performance [18,19]. SEM images of BiVO_4_, 1% Mo-BiVO_4_ and 3% Mo-BiVO_4_ are displayed in Figure 4a–c. For the reference of undoped BiVO_4_, the diameter is less than 200 nm, with many pores presenting on the surface (Figure 4a). With the addition of 1% of (NH_4_)_6_Mo_7_O_24_·4H_2_O, there is apparent change in morphologies, which are composed of closely arranged particles (Figure 4b). By further increasing the amount of (NH_4_)_6_Mo_7_O_24_·4H_2_O to 3%, some are broken and grown into large particles (Figure 4c). The surface area of three samples decreased with increasing Mo concentration, which is in agreement with SEM analyses (Figure 5). The above morphology transformation indicates that the introduction of Mo ions is beneficial for particle growth, which is in agreement with XRD results [20]. The breakage of some structure for 3% Mo-BiVO_4_ suffers from poor electron transportation, resulting in decreased photocatalytic enhancement. To further analyze the microstructure of Mo doped BiVO_4_ catalysts in detail, Figure 4d–f presents the TEM images of three products. When Mo is doped into BiVO_4_, the pores disappeared and the particles become bigger, which is in agreement with above results. Figure 4h–j shows the HR-TEM patterns of those samples. From the image of Figure 4h, the observation of clear lattice fringe spacing of 0.467 nm is indexed to the (011) crystal plane of BiVO_4_. As for 1% Mo-BiVO_4_ (Figure 4i), the obtained lattice fringes of d = 0.317 nm, 0.467 nm, and 0.307 nm are corresponded to the (130), (011) and (121) crystallographic planes of BiVO_4_, respectively. No Mo element fringes can be observed. This could be ascribed to the substitution Mo^6+^ ions in lieu of V^5+^, resulting in the formation of solid solution because of the similar ionic radius [21]. However, for 3% Mo-BiVO_4_ (Figure 4j), the lattice distance of d = 0.336 nm ascribed to (111) plane of MoO_3_ is measured near the edge of BiVO_4_, which coincides with XRD and XPS analysis. In effect, this phenomenon is very common in impurity doping research [22]. Namely, high concentrations of doped impurity elements easily combine with oxygen to form secondary oxides and then hinder the photocatalytic activity. The formation of MoO_3_ at the BiVO_4_ surface from excess dopants can explain the contradiction of higher doping concentration and higher crystallinity, which coincides with XRD analysis.

**Scheme 1:**
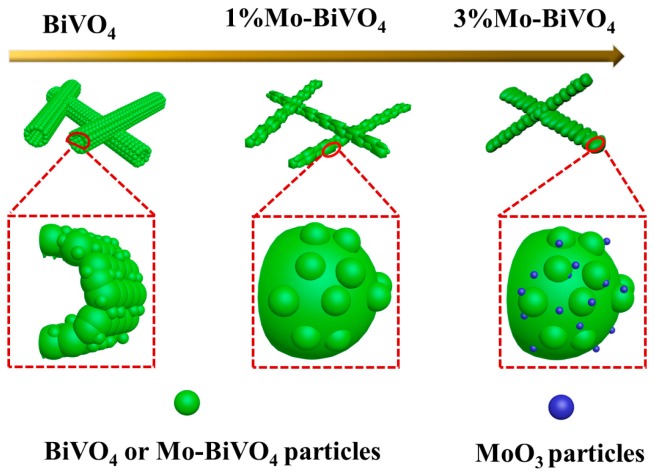
Illustration of the formation of different concentration Mo-BiVO_4_.

Based on the above analysis, we proposed a mechanism to explain the formation of the Mo-BiVO_4_, as shown in Scheme 1. Bare BiVO_4_ consists of homogeneous dispersed sol-gel particles with many pores. With a small amount of Mo ions doped into BiVO_4_ lattices, the BiVO_4_ particles become bigger while the pores disappear. No lattice fringe about Mo compounds can be detected, indicating the formation of solid solution. When the doping concentration reaches 3%, the saturated Mo ions preferably form MoO_3_ particles near the BiVO_4_ surface.

**Figure 6:**
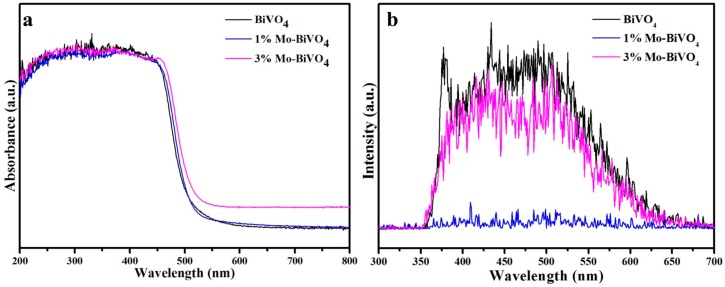
The UV-vis diffuse reflectance spectra (**a**); and PL spectra (**b**) of undoped BiVO_4_, 1% Mo-BiVO_4_ and 3% Mo-BiVO_4_.

Figure 6a shows the UV-vis diffuse reflectance spectra of all three samples. Distinctly, all three samples exhibit sharp absorption edges, in agreement with typically reported monoclinic BiVO_4_ [23,24]. Moreover, the UV-vis diffuse reflectance spectra curves of undoped BiVO_4_ and 1% Mo-BiVO_4_ almost completely overlap, there is little shift towards longer wavelength (red-shift) for 3% Mo-BiVO_4_, indicating that Mo dopants have no significant effect on the light absorption for BiVO_4_ structure. For a deep investigation, the PL spectra of all three samples were obtained to characterize the separation efficiency of the photogenerated electrons and holes. As shown in Figure 6b, both BiVO_4_ and 3% Mo-BiVO_4_ possess strong emission intensity in the range 375–550 nm. In theory, the PL emission is generated from the recombination of electrons and holes. However, the emission intensity of 1% Mo-BiVO_4_ is very weak and almost negligible. It is well known that lower PL intensity indicates higher separation efficiency, which could lead to a higher photocatalytic activity [25].

**Figure 7:**
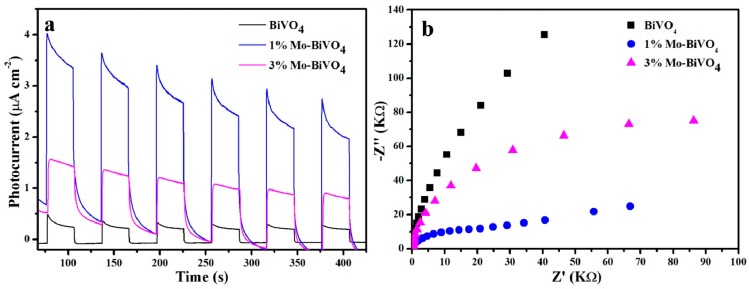
Photocurrent response (**a**); and EIS spectra (**b**) of undoped BiVO_4_, 1% Mo-BiVO_4_ and 3% Mo-BiVO_4_.

Finally, the photoelectrochemical behaviors of undoped BiVO_4_, 1% Mo-BiVO_4_ and 3% Mo-BiVO_4_ samples were performed. The photocurrent response results of three samples set applied potential as 0 are shown in Figure 7a. Obviously, 1% Mo-BiVO_4_ exhibits higher photocurrent intensity than pure BiVO_4_ and 3% Mo-BiVO_4_. The photocurrent of 1% Mo-BiVO_4_ is 3.5 times and 2.3 times higher than those of undoped BiVO_4_ and 3% Mo-BiVO_4_, indicating that lower concentration of Mo doped BiVO_4_ can obtain stronger ability in separation and transfer of photogenerated electron–hole pairs comparing with undoped or higher Mo doped BiVO_4_. Electronic impedance spectroscopy (EIS) is another important means to characterize the ability of charge transfer. As shown in Figure 7b, the order of arc radii is as follows: 1% Mo-BiVO_4_ > 3% Mo-BiVO_4_ > undoped BiVO_4_. It is well-known that a lower arc radius implies a higher efficient charge carrier transfer at the electrolyte and photoanode interface [26]. Therefore, 1% Mo-BiVO_4_ shows the strongest separation efficiency of electron–hole pairs, which is a key factor for high photocatalytic photocatalyst.

The foregoing analysis shows that the enhancement of photocatalytic activity for Mo doped in BiVO_4_ is ascribed to the improved electronic conductivity and the efficient separation of photogenerated carries. However, an increase in doped-Mo species is not accompanied by an increase in photocatalytic activities and photocurrent intensity. We proposed three main reasons to explain this phenomenon: (1) the occurrence of tetragonal phase for 3% Mo-BiVO_4_ can hinder the photocatalytic activity; (2) the formation of MoO_3_ at the surface of BiVO_4_ in 3% Mo-BiVO_4_ can increase the crystallinity and thereby reduce the crystal defects; and (3) the excess dopants may act as recombination centers [27].

## 4. Conclusions

In summary, photocatalysts with Mo doping into BiVO_4_ were successfully fabricated. The photocatalytic tests indicate that the 1% Mo-BiVO_4_ exhibits the strongest photocatalytic activity which can degrade 88.57% of MB within 120 min. For 1.5% Mo-BiVO_4_ and 3% Mo-BiVO_4_ photocatalysts, their photocatalytic performance is similar to the undoped BiVO_4_ with the exception of the strongest dark adsorption ability. This phenomenon should be due to transformation phase and the newly formed MoO_3_ particles on BiVO_4_ surface. The optical performance results indicate that the obvious enhancement of photocatalytic activity for 1% Mo-BiVO_4_ should be attributed to the efficient separation of photogenerated carries and improved electronic conductivity.

## Figures and Tables

**Table 1:** Binding energies of elements in the samples determined by high-resolution XPS analysis.

Binding Energy (eV)							
Samples	Bi 4f_7/2_	Bi 4f_5/2_	V 2p_3/2_	V 2p_1/2_	O 1s	M 3d_5/2_	Mo 3d_3/2_
BiVO_4_	159.2 ± 0.1	164.4 ± 0.1	516.7 ± 0.1	524.3 ± 0.1	529.8 ± 0.1	-	-
1% Mo-BiVO_4_	159.3 ± 0.1	164.6 ± 0.1	516.8 ± 0.1	524.5 ± 0.1	530.0 ± 0.1	232.3 ± 0.1	235.5 ± 0.1
3% Mo-BiVO_4_	159.5 ± 0.1	164.8 ± 0.1	517.0 ± 0.1	524.6 ± 0.1	530.2 ± 0.1	232.5 ± 0.1	235.7 ± 0.1
